# Rapid, Accurate, and Non-Invasive Measurement of Zebrafish Axial Length and Other Eye Dimensions Using SD-OCT Allows Longitudinal Analysis of Myopia and Emmetropization

**DOI:** 10.1371/journal.pone.0110699

**Published:** 2014-10-21

**Authors:** Ross F. Collery, Kerry N. Veth, Adam M. Dubis, Joseph Carroll, Brian A. Link

**Affiliations:** 1 Department of Cell Biology, Neurobiology and Anatomy, Medical College of Wisconsin, Milwaukee, Wisconsin, United States of America; 2 Department of Ophthalmology, Medical College of Wisconsin, Milwaukee, Wisconsin, United States of America; 3 Department of Biophysics, Medical College of Wisconsin, Milwaukee, Wisconsin, United States of America; University of Iowa, United States of America

## Abstract

Refractive errors in vision can be caused by aberrant axial length of the eye, irregular corneal shape, or lens abnormalities. Causes of eye length overgrowth include multiple genetic loci, and visual parameters. We evaluate zebrafish as a potential animal model for studies of the genetic, cellular, and signaling basis of emmetropization and myopia. Axial length and other eye dimensions of zebrafish were measured using spectral domain-optical coherence tomography (SD-OCT). We used ocular lens and body metrics to normalize and compare eye size and relative refractive error (difference between observed retinal radial length and controls) in wild-type and *lrp2* zebrafish. Zebrafish were dark-reared to assess effects of visual deprivation on eye size. Two relative measurements, ocular axial length to body length and axial length to lens diameter, were found to accurately normalize comparisons of eye sizes between different sized fish (R^2^ = 0.9548, R^2^ = 0.9921). Ray-traced focal lengths of wild-type zebrafish lenses were equal to their retinal radii, while *lrp2* eyes had longer retinal radii than focal lengths. Both genetic mutation (*lrp2*) and environmental manipulation (dark-rearing) caused elongated eye axes. *lrp2* mutants had relative refractive errors of −0.327 compared to wild-types, and dark-reared wild-type fish had relative refractive errors of −0.132 compared to light-reared siblings. Therefore, zebrafish eye anatomy (axial length, lens radius, retinal radius) can be rapidly and accurately measured by SD-OCT, facilitating longitudinal studies of regulated eye growth and emmetropization. Specifically, genes homologous to human myopia candidates may be modified, inactivated or overexpressed in zebrafish, and myopia-sensitizing conditions used to probe gene-environment interactions. Our studies provide foundation for such investigations into genetic contributions that control eye size and impact refractive errors.

## Introduction

Emmetropization is the process of correctly regulating eye globe size so that it matches the dioptric power of the anterior ocular structures, resulting in a sharply focused retinal image. This process requires tight control of the axial length of the eye, since an axial length longer than the focal length of the lens results in myopia (nearsightedness), while an axial length shorter than the focal length leads to hyperopia (farsightedness). Axial length, comprising the cornea, aqueous, lens, vitreous, retina and retinal pigment epithelium (RPE), is the largest contributor to refractive error leading to myopia [1] and is one of the most useful individual metrics used to assess myopia in humans. Homeostasis of axial length is controlled by regulated eye growth and subtle remodeling of ocular shape.

Myopia is the most common visual disorder in the world [2], affecting over 25% of people over 40 in the US and western Europe [3]. Prevalence rates are even higher in regions of Asia, where myopia approaches epidemic levels [4], as well as in certain ethnic populations from Indonesia and Japan [5], [6]. In addition to defocus of vision, myopia is also associated with pathologies including increased incidence of glaucoma, retinal detachment, cataracts, chorioretinal atrophy, scleral thinning, staphyloma, and damage to Bruch's membrane caused by choroidal thinning [3], [7]–[10].

The genetic causes of myopia are complex, with a large number of gene associations [11]. Genome-wide association studies have provided insights into the spectrum of candidate genes causing myopia in humans [12], [13], and have added to the considerable number of genes found to be modified by mutation or expression levels in myopes [11], [14]. Pathway analyses of the genes associated and altered in myopia suggests defects in the visual cycle/retinoid homeostasis, inner retinal neuron signaling, and regulation of structural components of the sclera and choroid [12], [13]. Much remains unknown about how these pathways interact to precisely regulate emmetropization.

Environmental factors and proper visual cues also play a role in emmetropization. Form-deprivation of vision in young children leads to myopic phenotypes [15], [16]. Excessive near-work such as reading or computer work has been previously proposed as a risk factor, though further studies have found the potential for association to be weak [17], [18]. Conversely, time spent outdoors is associated with reduced incidences of myopia [19], [20].

Investigation of genetic or environmental causes of myopia and the extent of their contribution requires isolation of each potential factor, as well as comparison with an appropriate control group. Animal models have provided valuable information on how individual genes are associated with myopic eye growth [21]–[23] [24], while form-deprivation using light-diffusing goggles or eyelid suturing [25], [26] [27], [28], or inducing optical defocus by applying positive or negative lenses have demonstrated the importance of correct visual cues to instruct emmetropic eye growth [29]–[31]. Dark-rearing is also used to induce refractive error, though interestingly, the direction of ametropia (hyperopia *vs* myopia) following dark-rearing has been found to differ between species, and the direction of refractive error induced may depend on whether the animal is visually experienced before the onset of dark-rearing [32], [33].

Zebrafish (*Danio rerio*) as an experimental model offer many advantages for vision research. They are diurnal and have tetrachromatic color vision [34] with a cone-rich retina and therefore model the high reliance on cone-mediated vision of humans. Assessment of visual function, including electroretinography, behavioral response to visual stimuli, and learned responses to training have been well demonstrated in zebrafish [35], [36]. Genetic modifications can be carried out with relative ease, with knock-in of transgenes facilitated by transposon-based recombineering [37], and genome editing made possible by TALEN and CRISPR/Cas technologies [38], [39]. Promoter tools that direct expression uniquely in the sclera [40], cones [41], rods [42], Müller glia [43], horizontal cells [44], amacrine cells [45], bipolar cells [46], retinal ganglion cells [47], or lens of the eye [48] have been characterized. Thus, the amenability of zebrafish to genome manipulation will facilitate mutant lines that can be screened rapidly for changes in eye axial length or other eye metrics using the methods outlined here.

In this study, we describe and validate methodology to use SD-OCT with zebrafish in order to study myopia-associated phenotypes. We then characterized growth over time of wild-type and large-eyed *lrp2* mutants, using these non-invasive methods. Finally we demonstrate that zebrafish, like other species, show abnormal axial length growth when subjected to constant darkness. Cumulatively, these studies provide a framework for probing the genetic contributions to eye growth and remodeling, particularly as it relates to myopia.

## Materials and Methods

### Ethics statement

This study was carried out in strict accordance with the recommendations in the Guide for the Care and Use of Laboratory Animals of the National Institutes of Health. The protocol was approved by the Institutional Animal Care and Use Committee of the Medical College of Wisconsin, protocol number AUA1378. Zebrafish from 7 dpf to 2 years of age were anesthetized using tricaine methanesulfonate at 0.016% buffered to pH = 7.2, and all efforts were made to minimize suffering.

### Zebrafish maintenance

Zebrafish (Danio rerio) were maintained at 28.5°C on an Aquatic Habitats recirculating filtered water system (Aquatic Habitats, Apopka, FL) in reverse-osmosis purified water supplemented with Instant Ocean salts (60 mg/l) on a 14 h light: 10 h dark lighting cycle and fed a standard diet [49]. For dark-rearing, a cohort of sibling wild-type zebrafish larvae was visually experienced until 5 days post-fertilization (dpf) then divided into two groups of tanks with one group receiving normal cyclic lighting and the other wrapped in aluminum foil to block entry of light. Sibling cohorts in either dark-rearing tanks or light-reared control tanks were fed brine shrimp only. At 1 month, 3 months and 4.5 months following the onset of dark-rearing, tanks were unwrapped and immediately analyzed by OCT for eye measurements, along with the corresponding light-reared group. Once exposed to light, no dark-reared fish were used for measurements at subsequent timepoints.

### Spectral domain-optical coherence tomography (SD-OCT)

Zebrafish from 7 dpf to 2 years of age were anesthetized as outlined above and placed on the imaging stage. Axial length, lens diameter and retinal radius were measured for populations of zebrafish at 7 dpf, 15 dpf, 1 month, 2 months, 3 months, 6 months, 1 year, 1.5 years and 2 years of age. At least 20 eyes (2 eyes per fish from 10 fish) were measured per timepoint. However, because of rapid eye growth and higher variability in ocular size during early and juvenile development, eyes from 20 fish at 7 dpf, 15 dpf and 1 month were measured, giving 40 data points at these times. At selected timepoints, the same measurements were taken of *lrp2* zebrafish, which have a premature truncation in the gene encoding endocytic receptor Lrp2, leading to enlarged eye size and high myopia [22], [50]. Both eyes from individual fish were regarded as separate data points, as in other studies relating to eye morphology [51].

Zebrafish eyes were imaged using a Bioptigen Envisu R2200 SD-OCT imaging system with a 12 mm telecentric lens (Bioptigen, Morrisville, NC) using a Superlum Broadlighter T870 light source centered at 878.4 nm with a 186.3 nm band width (Superlum, Cork, Ireland). Acquisition settings were controlled with the InVivoVue software platform and eye measurements made using the built-in manual caliper tool within the program. Eyes were imaged from a 100 B-scans covering a 3 mm×3 mm field with 700 A-scans per B-scan following orientation so that opposing sides of the iris were centered in both horizontal and vertical planes to ensure the central axial length of the eye was measured. To calibrate images in the medial dimension, 6-month-old zebrafish were imaged by SD-OCT while an refractive index constant was varied in the acquisition software. Zebrafish were then euthanized and their lenses enucleated and immediately measured by placing the lens beside a scaled calibration slide and viewing with a dissecting microscope. We found that wild-type zebrafish need a refractive index constant of 1.30 (±0.03, standard deviation) applied in the software to correct the aspect ratio of the produced image. This constant was used in all subsequent measurements (Figure S1). Representative images of B-scans were cropped using Adobe Photoshop and annotated using Adobe Illustrator (Adobe Systems Incorporated, San Jose, CA).

### Eye and body length measurement

Zebrafish eye dimensions were measured as follows: axial length – front of cornea to back of RPE; lens diameter – anterior surface of lens to posterior surface; retinal radius – center of lens to the back of the RPE. We have found that the hyper-reflectivity in the RPE is caused by melanin by comparing pigmented and non-pigmented RPE samples side by side (data not shown). For eyes larger than 1.7 mm axial length, B-scan images were adjusted so that the inverted image of the anterior segment appeared along with the real image of the retina [52] (also referred to as the mirror artifact [53]). The eye was then visualized and measured in two parts – from the corneal surface to the top of the viewing window (path-matching position or 0-delay line), and from the back of the RPE to the top of the window. The distances measured in these two parts were summed to give the full axial length. Body length was measured from the tip of the head to the end of the trunk (before the caudal fin) using a ruler. Relative refractive error was calculated as 1- (retinal radius/F), where F, an idealized focal length = lens radius × 2.324, using a coefficient extrapolated from a large population group (n = 240) plot of lens radius *vs* retinal radius (see Results). Relative refractive error values are unitless, with values lower than zero indicating that the eye is myopic (or that the observed distance from lens center to RPE is greater than the expected retinal radius) and values greater than zero indicating that the eye is hyperopic (or that the observed distance from lens center to RPE is less than the expected retinal radius). The relative refractive error is based on the following assumptions: 1. that the refractive index of the fish lens is constant, and 2. that the distance from the center of the lens to the RPE is equal to the focal length of the lens of wild type fish.

### Laser-assisted raytracing of focal length

Six-month-old zebrafish were euthanized following SD-OCT and lenses dissected into phosphate-buffered saline. Lenses were placed on a modeling-clay pedestal in a chamber containing phosphate-buffered saline with a drop of milk added to aid in laser-beam visualization. Lenses were oriented with the aid of the lens zonules so that the laser passed through the lens perpendicular to the dorsal-ventral axis. The chamber was placed under a dissecting microscope and a red laser pointer (wavelength: 630–680 nm; max. output <5 mW) positioned so that the beam passed through the center of the lens. A micromanipulator was used to move the laser pointer so that rays passed through the lens at multiple points, with images taken at each point. Rays were traced using ImageJ (Rasband, W.S., ImageJ, U. S. National Institutes of Health, Bethesda, Maryland, USA, http://rsb.info.nih.gov/ij/) and merged using the maximum intensity projection feature. Focal length was measured from the center of the lens to the point where the rays converge. Lens dimensions were measured at the same time. Scaling was carried out by imaging a microscope calibration slide at the same magnification. Ray-tracing was carried out on wild-type and *lrp2* mutant lenses; however, dark-reared zebrafish lenses were not measured by ray-tracing.

### Statistical analysis

Eye measurements were processed using Microsoft Excel (Microsoft, Redmond, WA) and graphed using GraphPad Prism (GraphPad, La Jolla, CA). Standard error of means (SEM), standard deviation (SD), analysis of variance (ANOVA) with post-test analyses, and regression analyses were calculated using GraphPad Prism. In all figures, significance levels are defined as follows: ns, p>0.05; *p≤0.05; **p≤0.01; ***p≤0.001; ****p≤0.0001.

## Results

### SD-OCT allows in vivo imaging of the growing and mature zebrafish eye

**Figure 1:**
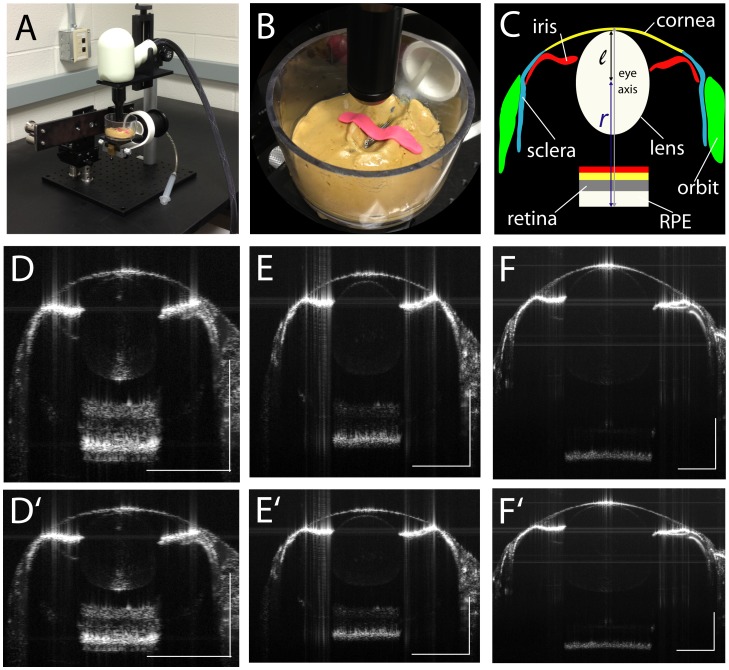
SD-OCT imaging stage and B-scan examples. A. Imaging stage for Bioptigen Envisu 2200 with zebrafish immersion cuvette. B. Zebrafish immobilized using a strip of modeling clay to prevent movement or floating during immersion. C. Schematic showing highly reflective structures of the zebrafish eye traced over 1 mpf B-scan; l, lens radius; r, retinal radius. D. 15 dpf; E. 1 mpf; F. 2 mpf. Scale bars: 300 µm. D′, E′, F′: as above with aspect ratio corrected to 1∶1. As zebrafish eyes age and increase in size, the reflected signal from the retina is reduced, making lamination less visible, though the strongly hyper-reflective RPE can still be observed.

Zebrafish eyes grow continuously during their lifetime. Though the cornea of the zebrafish eye has a negligible contribution to refraction owing to its aquatic environment, measurements of axial length (from anterior cornea to RPE) provide information on the size of the eye that may be useful when considering refractive error in other animal species that have an air-to-cornea interface, and therefore a corneal role in refraction. To demonstrate the utility of OCT imaging of zebrafish eyes from early development to full adulthood, we show representative images (Figure 1). In each case, the anatomy of the structures of the eye can be seen clearly, including the cornea, sclera, iris, lens and laminated retina. At 15 dpf, the lamination of the retina can be clearly seen (Figure 1 D, D′), though at 1 mpf, only the RPE and GCL can be seen (Figure 1 E, E′). At 2 mpf and older, only the RPE can be seen (Figure 1 F, F′). This reduction in reflective signal is likely due to the greater distance traveled by the light during imaging as eye size increases, and light absorption by the vitreous. Figure 1 C shows a schematic of the 1 month eye in Figure 1 E to aid in orientation. The spherical zebrafish lens appears oval since the light rays used to acquire images are themselves refracted as they traverse the lens. This phenomenon, however, does not affect the reliability of the SD-OCT measurements. Images where the aspect ratio has been converted to 1∶1 with the aid of scale bars to represent the true dimensions of the zebrafish eye are also shown (Figure 1 D′, E′, F′). When comparing anterior-posterior and proximal-distal axis measurements of isolated wild-type lenses, the degree of circularity was 0.98 (±0.03, standard deviation), and *lrp2* lenses had a degree of circularity of 0.99 ((±0.02, standard deviation), where a perfect circle score equals 1.00 (Figure S1).

### Accurate, longitudinal measurements of eyes using SD-OCT

**Figure 2:**
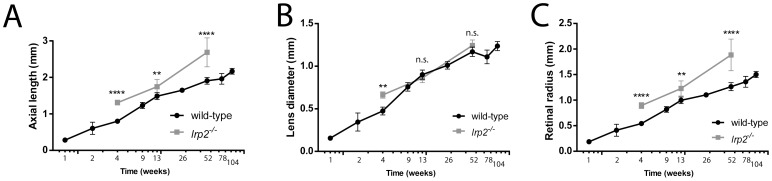
Zebrafish eye metrics graphed with respect to time. A. Eye axial length measured by SD-OCT increases during the lifetime of the growing fish. *lrp2* zebrafish eyes are larger than wild-type at all timepoints measured. B. Lens diameter increases as the fish grows. *lrp2* zebrafish lenses are larger than wild-type at 1 month, but are not significantly larger at 3 months or 1 year. C. Retinal radius increases consistently as the fish age, and *lrp2* retinal radii are greater than wild-type at all timepoints measured. Error bars show SD.

Zebrafish eye axial length, lens diameter and focal distance all increased over time (Figure 2 A, B, C; Figure S2). Growth for all three parameters was most rapid during the first 3 months of age, and then slowed, but continued to increase. Eyes of *lrp2* mutant fish were measured by SD-OCT and, in agreement with previously described histological data, *lrp2* mutant eyes were found to have greater and more rapidly increasing axial lengths and retinal radii than wild-type zebrafish. Lens diameter was greater in *lrp2* mutant fish at 1 mpf, but did not differ significantly from wild-type values at other timepoints measured.

### Zebrafish body length and lens diameter can be used to normalize eye size

**Figure 3:**
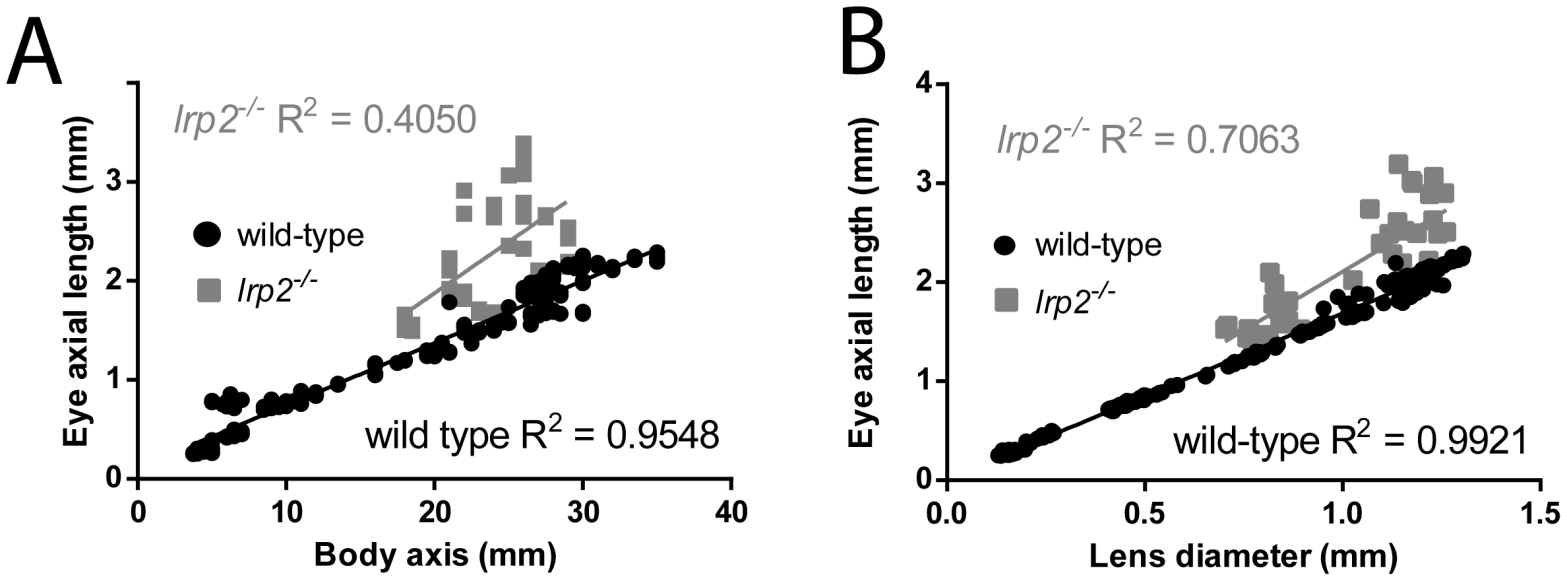
Eye size measurements can be normalized for comparison using body axis length or lens diameter. A. Eye axial length in wild-type zebrafish has a good linear relationship to body axis length with points close to the best-fit trendline. *lrp2* zebrafish axial length:body length ratios are mostly longer than wild-type, and show less correlation with the best-fit line. B. Eye axial length in wild-type zebrafish has an excellent linear relationship with lens diameter, while *lrp2* zebrafish axial length:lens diameter ratios show less correlation with the best-fit line. C. Images of wild-type and *lrp2* mutant zebrafish at 2 mpf. At this age, the *lrp2* enlarged eye phenotype is not always obvious, and one fish can have emmetropic eye growth (*lrp2* (normal eye size)) while another has enlarged, myopic eyes, with the left eye more affected than the right (*lrp2* (large eye size)). OCT images of each eye are shown. D. Measurements taken from OCT images for each eye in C, as well as relative refractive error showing that only the *lrp2* (large eye size) fish is myopic at this time.

In order to assess relative eye sizes uniformly both within populations and between different populations, such as mutant *vs* wild-type comparisons, or drug-treated *vs* control groups, we looked to express the axial length of the eye as a function of another, independent parameter. We tested the effectiveness of body axis length, and lens diameter as metrics for normalization. Graphing individual eye axial lengths with respect to either body axis or lens diameter and applying linear regression calculations showed that both potential normalizing factors yielded good coefficients of determination (eye axis:body axis; R^2^ = 0.9548, p<0.0001; eye axis:lens diameter: R^2^ = 0.9921, p<0.0001; Figure 3 A, B). These data indicated that for zebrafish, both eye axis:body length and eye axis:lens diameter ratios faciliate normalization of eye size between comparison groups. When normalizing with these metrics for *lrp2* eye axial lengths, eye axis:body length and eye axis:lens diameter ratios were both consistently higher than wild-types, demonstrating that axial lengths are greater in *lrp2* mutant eyes. Coefficients of determination for eye axis:body length and eye axis:lens diameter were lower for *lrp2* eyes than wild-types (R^2^ = 0.4050, p<0.0001; R^2^ = 0.7063, p<0.0001, respectively), likely reflecting the variability in *lrp2* eye size. We cannot, however, rule out the possibility that *lrp2* mutants have different eye axis:body length or eye axis:lens diameter relationships that may contribute to the lower coefficients of determination.

### Zebrafish lens size is correlated with retinal radius with a linear constant, which can be used to calculate relative refractive error in experimental groups

**Figure 4:**
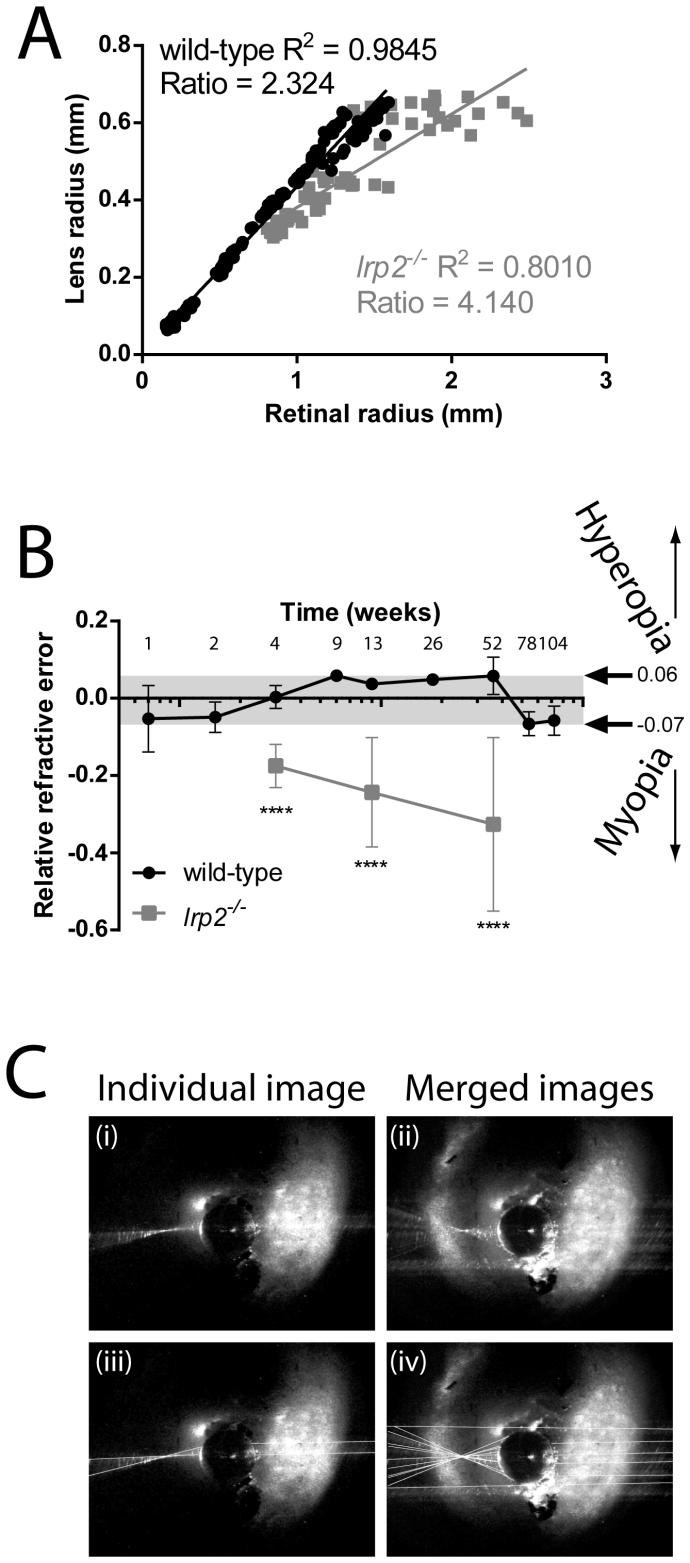
SD-OCT used to measure the relationship between zebrafish retinal radius and lens radius, or relative refractive error. A. Wild-type zebrafish retinal radius shows a very good linear relationship with lens radius, and the best-fit line can be used to calculate the ratio coefficient which predicts correct retinal radius for each eye from the measured lens radius. *lrp2* retinal radius: lens radius ratios have poorer linear relationships and have a higher ratio coefficient predicting that each *lrp2* eye will have a longer required focal distance than wild-type. B. Relative refractive error compares the difference between calculated retinal radius based on lens radius measurements and observed retinal radius (center of lens to back of RPE). Relative refractive errors are made from a general equation, and individual wild-type sample groups are shown at each timepoint to demonstrate the variability seen when using this equation (grey bar shows lowest and highest average values). *lrp2* zebrafish are more myopic than wild-types and consistently become more myopic as they age. Error bars show SD. C. Example of laser-assisted ray-tracing through a dissected lens to calculate focal length. Shown are individual images (i, iii), or composites (ii, iv) to indicate the light-ray intersection point either without line tracing (i, ii), and with lines tracing (iii, iv) to aid visualization.

We plotted the lens radius against retinal radius of 240 zebrafish eyes at timepoints throughout the lifespan of the fish. This allowed us to determine a general constant, or coefficient, relating lens radius to retinal radius (lens-retinal radius coefficient), also known as Matthiessen’s ratio [54], [55] (Figure 4 A). Using this coefficient, we defined the expected retinal radius for wild-type fish with respect to lens size, and compared retinal radii for other fish with potential myopia or hyperopia to these values. We refer to this comparison as relative refractive error. We found that lens radius × 2.324 equaled the calculated retinal radius based on the equation of the best fit linear regression, with a strong coefficient of determination (R^2^ = 0.9845, p<0.0001). This number is nearly identical to the coefficient determined for goldfish [56]. Calculating the general lens-retinal radius coefficient for all *lrp2* eyes measured gave a value of 4.140, with a lower coefficient of determination (R^2^ = 0.8010, p<0.0001), showing that *lrp2* retinal radii are significantly greater than the values predicted from lens measurement. The lower coefficient of determination likely reflects the variability observed in *lrp2* eye size. At 1 month, *lrp2* zebrafish have greater negative relative refractive error than wild-type (−0.175 vs 0, respectively), and as they age, their relative refractive errors continue to become greater than wild-type controls (−0.244 vs 0.037 at 3 mpf, −0.327 vs 0.058 at 1 ypf) (Figure 4 B). To define normal axial length variability, individual wild-type zebrafish used to derive the relative refractive error showed a range of relative refractive errors from 0.06 to −0.07 (Figure 4 B, gray bar). Applying these normalization and relative refractive error calculations to sample wild-type and *lrp2* mutants at 2 mpf demonstrates that variability in mutant populations requires accurate measurements to assess myopic phenotypes (Figure 3 C, D).

### Optical ray-tracing shows wild-type zebrafish eyes have matching focal lengths and retinal radii, while retinal radii exceed focal lengths in myopic *lrp2* zebrafish

To verify that the focal length of the zebrafish lens correlated with the retinal radius, or distance from the center of the lens to the RPE measured using SD-OCT, we carried out ray-tracing on wild-type and *lrp2* mutant lenses to calibrate medial dimension measurements (Figure 4 C). Comparing measurements of ten wild-type lenses, the average distance of the RPE from the center of the lens as measured by SD-OCT was 0.995 mm±0.037 mm, while the ray-traced focal length was 1.080 mm±0.083 mm. The retinal radius calculated from lens radius and lens-retinal radius coefficient was 1.157 mm±0.043 mm. There were no significant differences in these values, validating OCT methods for calculating retinal radii. Importantly, the focal length in wild-type fish measured directly by lens refraction matches the retinal radius, strongly suggesting wild-type fish are emmetropic, and confirming retinal radius as a good proxy for focal length (Table 1). There was no difference between the ray-traced focal length and expected retinal radii (lens-radius derived) for ten *lrp2* fish of the same age. However, when comparing focal length, or expected retinal radius, to the observed position of the RPE from the lens center as measured by SD-OCT, *lrp2* fish were found to be myopic (SD-OCT measured retinal radius = 1.550 mm±0.437 mm; raytraced focal length = 1.228 mm±0.050 mm; expected retinal radius from lens radius and lens-retinal radius coefficient = 1.144 mm±0.057 mm).

**Table 1:** Two-way ANOVA with Tukey’s post-test analysis comparing methods of focal length and retinal radius measurement in wild-type and *lrp2* zebrafish eyes.

Focal length and retinal radius comparisons	Summary	Adjusted P Value	Mean Difference (mm)
wild-type raytraced vs. wild-type SD-OCT	ns	0.9104	0.085
*lrp2* raytraced vs. *lrp2* SD-OCT	**	0.0048	−0.322
*lrp2* raytraced vs. *lrp2* calculated	ns	0.9144	0.084
*lrp2* SD-OCT vs. *lrp2* calculated	***	0.0002	0.407

Multiplicity-adjusted p values are shown. No significant differences were seen when calculating focal length and retinal radius in wild-type zebrafish using raytracing of dissected lenses, measurement from center of lens to back of RPE using SD-OCT, or calculation from lens radius. However, measurements of focal length and retinal radius were significantly different for *lrp2* when comparing values measured by ray-tracing or by calculation from lens radius. This shows that the focal distance required by *lrp2* eyes is significantly longer than the focal length of the lens, and that they are myopic.

We also attempted to directly measure in vivo the degree of refractive error in wild-type zebrafish using retinoscopy. To do so, adult zebrafish were anesthesized and either placed out of water or immobilized in water and examined using a retinoscope. However, using a standard apparatus (standard streak retinoscope, Welch Allyn, NY), retinoscopy was not possible in zebrafish due to the small eye size and fixed pupil diameter.

### Zebrafish respond to dark-rearing by becoming myopic

**Figure 5:**
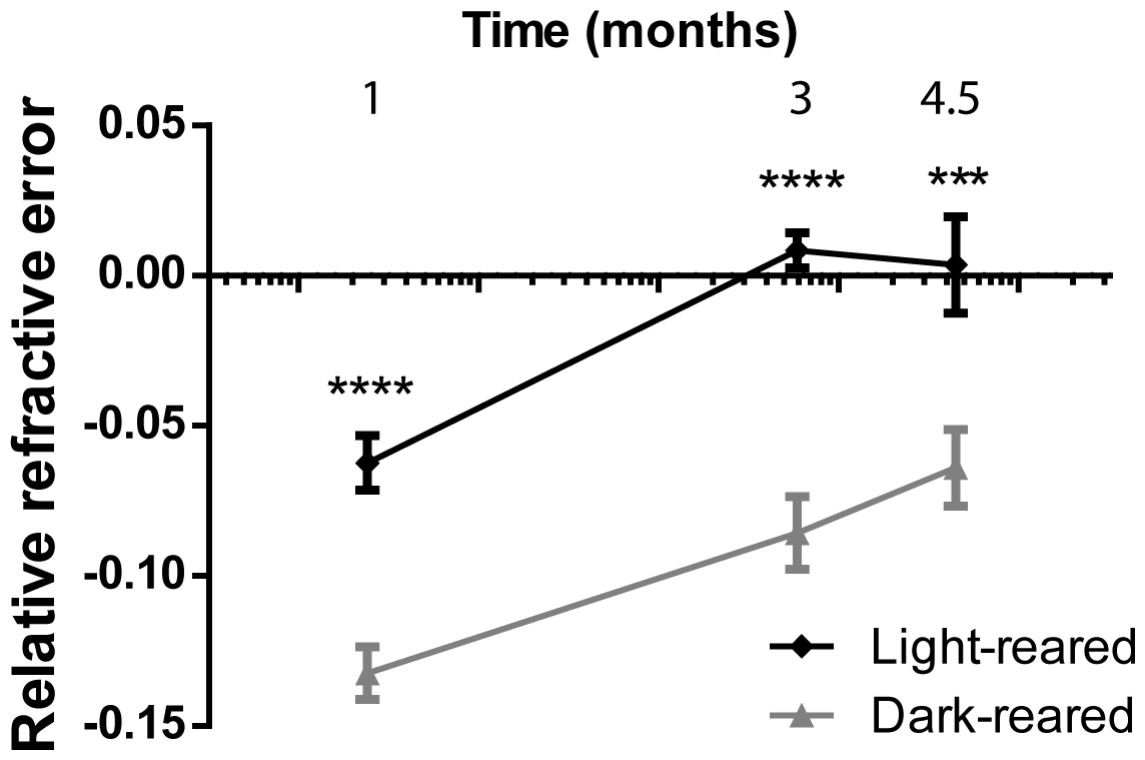
Dark-rearing zebrafish leads to myopia induction. Sibling zebrafish were raised in either light or dark conditions and relative refractive errors measured at various timepoints. Dark-reared zebrafish showed relative refractive errors at each timepoint, which were significantly lower than light-reared controls. Error bars show SEM.

Ametropia has been induced in several animal models by dark-rearing, where the lack of light deprives animals of an emmetropization input signal [57], [58]. At 1 month, dark-reared zebrafish were significantly more myopic than light-reared siblings (relative refractive errors of −0.062 for light-reared vs −0.132 for dark-reared) (Figure 5). Three month and 4.5 month dark-reared zebrafish were also significantly more myopic than their light-reared siblings (0.008 for light-reared vs −0.086 for dark-reared at 3 mpf, 0.004 for light-reared vs −0.064 for dark-reared at 4.5 mpf). Changes in retinal radius were caused by axial length elongation, as the lens diameters were only significantly different between age-matched light- and dark-reared zebrafish at 1 month (Table S1). Axial lengths and retinal radii that have not been normalized should be viewed with caution as they do not take the size of the animal into account. Nonetheless, at 1 month and 4.5 months, axial lengths and retinal radii are significantly longer in dark-reared fish than light-reared controls.

## Discussion

This study shows that SD-OCT can be used to rapidly and accurately measure the size of the zebrafish eye, lens and retinal radius during both emmetropic wild-type and myopic *lrp2* eye growth. Using SD-OCT, high quality images can be non-invasively acquired of developing and mature eyes and used to generate a representation of the interior of the eye. We show that the zebrafish eye, lens, and retinal radius grow rapidly during larval and juvenile development, then grow more slowly. By comparing lens dimensions and refractive properties in vivo and ex vivo, we show that SD-OCT measurements can be used to measure retinal radius, which correlates with focal length in wild-type fish, and to infer refractive error in the zebrafish eye. Comparing ray-traced focal length and retinal radius measured by SD-OCT showed no significant difference within a wild-type sample group. However, *lrp2* fish were found to have retinal radii significantly longer than predicted by the refractive power of their lenses, and are therefore myopic.

We have demonstrated methods to normalize the absolute size of the eye relative to two separate parameters, overall body size and lens diameter. In order to test whether an individual eye is the correct length to match the corresponding focusing power, or whether an eye is considered large relative to the overall body size of the subject, an invariant and independent metric must be used to normalize between test subjects. This is especially true when studying animals that may show considerable variability in size within a population, such as the zebrafish. Eye size has been shown in other species to be tightly controled as a function of overall body size [59], [60], yet many animal models with eye defects have abnormal body sizes [61]. For example in chickens, the retinopathy globe enlarged (RGE) model of myopia, has a smaller body size than wild-types. Normalization with a different parameter is therefore essential to demonstrate that RGE chickens have proportionally larger eyes [62]. Furthermore, global gene disruption or bath application of drugs that affect the body axis can make this ratio difficult to interpret in the context of refractive error (R. Collery, unpublished data). In short, two methods of eye size and retinal radius normalization will be valuable when manipulating myopia-associated genes and pathways that have pleiotropic functions in zebrafish.

Visual input is known to be critical for emmetropization, and we show that dark-rearing of wild-type zebrafish from early development leads to myopia. We note that while *lrp2* mutant zebrafish become more myopic as they age, dark-reared fish tend towards emmetropia over time, suggesting that innate, non-visual signals in the zebrafish eye also contribute to emmetropia.

Because SD-OCT is rapid and accurate, eye measurements can be used to quickly infer the degree of emmetropia. Establishing a simple equation relating lens radius to expected retinal radius will facilitiate rapid analysis of zebrafish genetic mutants for ametropia, as well as identification of genetic polymorphisms or small molecules that modify refractive error. We note that despite accurate measurement of dimensions of the zebrafish eye, we do not directly measure refractive error with the intact eyes of individual fish, though we have shown that SD-OCT can be used to measure components of the eye that define emmetropization in wild-type and *lrp2* myopic animals. Caveats to our method of calculation are that we assume that the refractive indices of lenses are the same between subjects, that refractive indices are constant within each lens, and that lens size (or body length, depending on the metric used for normalization) is unaffected in mutant or drug-treated zebrafish. Nevertheless, our measurements and calculation can robustly detect myopia that is either genetic (*lrp2*) or environmentally-induced (dark-rearing), and we propose that combining SD-OCT imaging with the zebrafish model system will facilitate investigation of candidate genes or environmental conditions causitive for myopia.

In conclusion, the zebrafish eye, with a non-accommodating spherical lens and non-contractile iris, combined with negligible contribution of corneal refraction due to their aquatic nature, makes zebrafish an excellent model system to isolate the mechanisms of axial length and lens radius on emmetropization. Since the largest contributor to myopic development is axial length, a model system that combines efficient genome editing, susceptibility to visual environment manipultion that alters eye growth, and is also readily accessible for pharmacologic experiments will be very useful in probing the genetic and cellular mechanisms underlying myopia.

## Supporting Information

Figure S1 **Ray-tracing analysis and comparison of focal length and retinal radius measurement.** A–E. Individual beams from a laser refracted by a dissected lens using different entry points. F. Merging A–E shows the intersection point of each ray as the focal length. A′–F′ show lines traced over the light rays in the images of A–F. G. Wild-type 6-month zebrafish lens measurements showing ten individual lens metrics and retinal radius and focal length values (2 per fish, 5 fish) along with averaged values and standard deviation error. H. Equivalent measurements of G. for age-matched *lrp2* lenses. I. t-test results comparing wild-type and *lrp2* lens dimensions, circularity, software refractive index correction factor and raytraced focal length. J. Comparison of methods of retinal radius measurement and prediction, using raytracing of dissected lenses, measurement from center of lens to back of RPE using SD-OCT, and calculation from lens radius for ten individual wild-type 6-month zebrafish eyes (2 per fish, 5 fish). K. as J, for age-matched *lrp2* eyes. L. t-test results comparing wild-type and *lrp2* lens and retinal radius and focal length measurements. Statistical analysis is shown in Table 1.
(TIF)

Figure S2 **Zebrafish eye and body parameters graphed with respect to time using a linear X-axis for time.** A. Eye axial length measured by SD-OCT increases during the lifetime of the growing wild-type fish in two phases. The first is rapid (labeled I, dark grey box, slope = 0.01326±0.0003736 (1/slope = 75)) and the second is slower (labeled II, light grey box, slope = 0.0009584±4.517e-005 (1/slope = 1043)). B. Lens diameter increases as the fish grows in two phases. The first is rapid (labeled I, dark grey box, slope = 0.1602±0.009701 (1/slope = 120)) and the second is slower (labeled II, light grey box, slope = 0.0004333±3.207e-005 (1/slope = 2308)). C. Retinal radius increases as the fish grows in two phases. The first is rapid (labeled I, dark grey box, slope = 0.008863±0.0002589 (1/slope = 113)) and the second is slower (labeled II, light grey box, slope = 0.0007279±3.085e-005 (1/slope = 1374)).
(TIF)

Table S1 A. Comparison of axial length, lens radius and retinal radius at 1 month, 3 months and 4.5 months between light-reared and dark-reared zebrafish. Values show average measurements ± SD. B. t-test results comparing axial length, lens radius and retinal radius for significant differences.
(TIF)
