# Correction: Rapid, Accurate, and Non-Invasive Measurement of Zebrafish Axial Length and Other Eye Dimensions Using SD-OCT Allows Longitudinal Analysis of Myopia and Emmetropization

**DOI:** 10.1371/journal.pone.0119779

**Published:** 2015-03-30

**Authors:** The PLOS ONE Staff

There is an error in Fig. 3. Panels C and D were inadvertently omitted. The authors have provided the corrected figure below.

**Fig 3:**
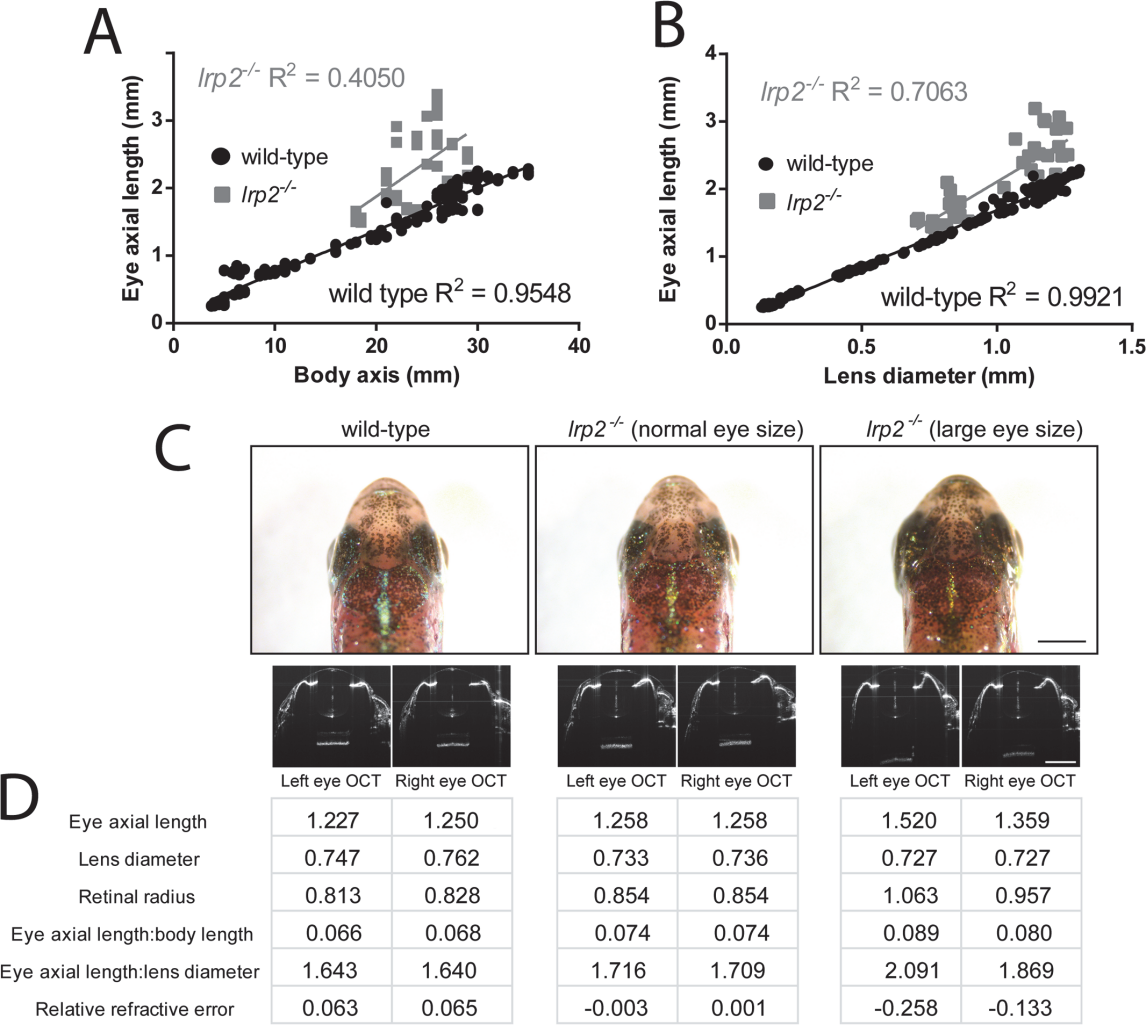
Eye size measurements can be normalized for comparison using body axis length or lens diameter. A. Eye axial length in wild-type zebrafish has a good linear relationship to body axis length with points close to the best-fit trendline. *lrp2* zebrafish axial length:body length ratios are mostly longer than wild-type, and show less correlation with the best-fit line. B. Eye axial length in wild-type zebrafish has an excellent linear relationship with lens diameter, while *lrp2* zebrafish axial length:lens diameter ratios show less correlation with the best-fit line. C. Images of wild-type and *lrp2* mutant zebrafish at 2 mpf. At this age, the *lrp2* enlarged eye phenotype is not always obvious, and one fish can have emmetropic eye growth (*lrp2* (normal eye size)) while another has enlarged, myopic eyes, with the left eye more affected than the right (*lrp2* (large eye size)). OCT images of each eye are shown. D. Measurements taken from OCT images for each eye in C, as well as relative refractive error showing that only the *lrp2* (large eye size) fish is myopic at this time.
