# Supplementary figures and images for: Rapid, Accurate, and Non-Invasive Measurement of Zebrafish Axial Length and Other Eye Dimensions Using SD-OCT Allows Longitudinal Analysis of Myopia and Emmetropization
Source: PLoS One. 2014 Oct 21;9(10):e110699. doi: 10.1371/journal.pone.0110699 (PMC4205002; doi:10.1371/journal.pone.0110699)

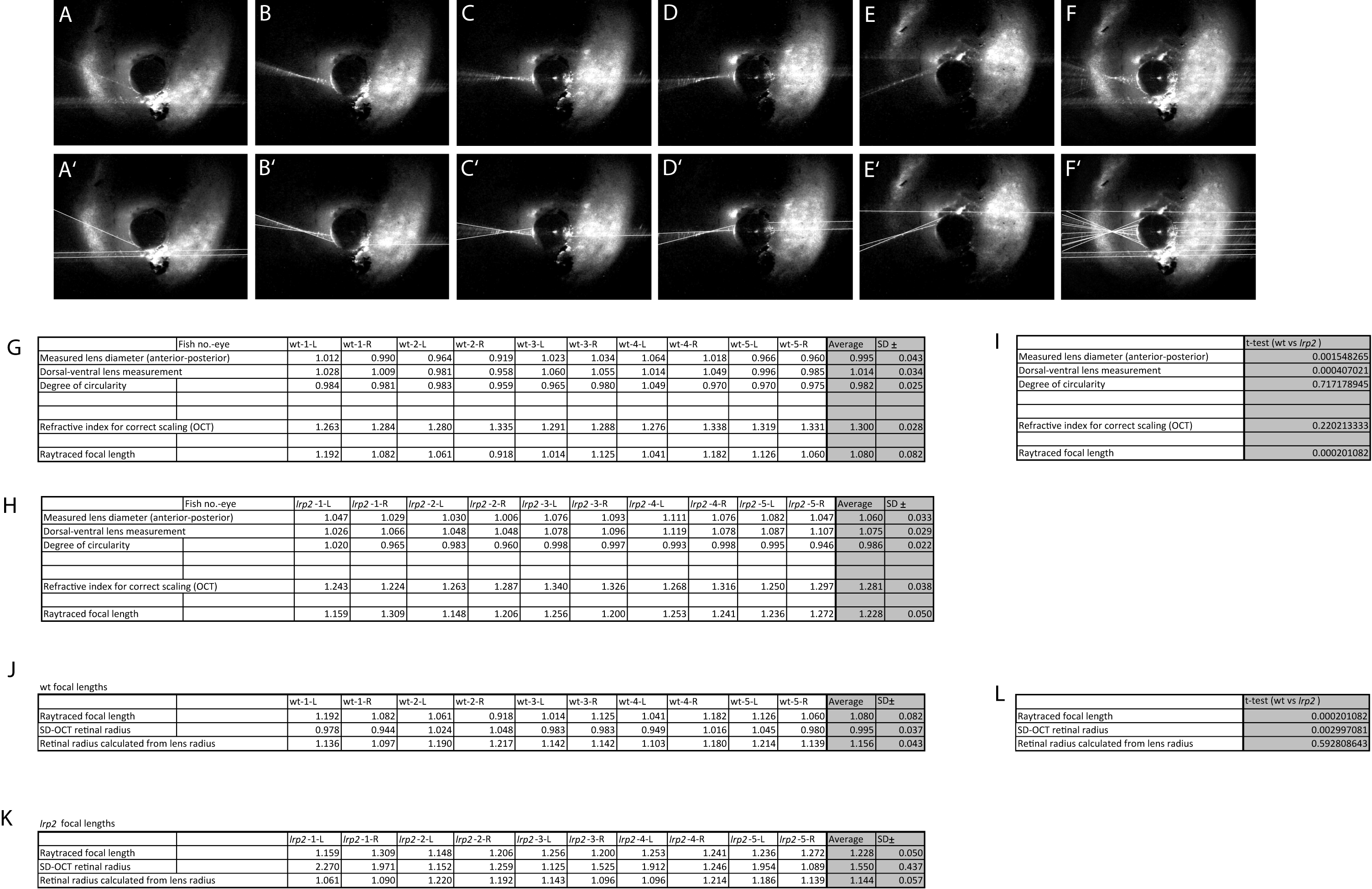

Supplement: Figure S1 — Ray-tracing analysis and comparison of focal length and retinal radius measurement. A–E. Individual beams from a laser refracted by a dissected lens using different entry points. F. Merging A–E shows the intersection point of each ray as the focal length. A′–F′ show lines traced over the light rays in the images of A–F. G. Wild-type 6-month zebrafish lens measurements showing ten individual lens metrics and retinal radius and focal length values (2 per fish, 5 fish) along with averaged values and standard deviation error. H. Equivalent measurements of G. for age-matched lrp2 lenses. I. t-test results comparing wild-type and lrp2 lens dimensions, circularity, software refractive index correction factor and raytraced focal length. J. Comparison of methods of retinal radius measurement and prediction, using raytracing of dissected lenses, measurement from center of lens to back of RPE using SD-OCT, and calculation from lens radius for ten individual wild-type 6-month zebrafish eyes (2 per fish, 5 fish). K. as J, for age-matched lrp2 eyes. L. t-test results comparing wild-type and lrp2 lens and retinal radius and focal length measurements. Statistical analysis is shown in Table 1. (TIF) [file pone.0110699.s001.tif]

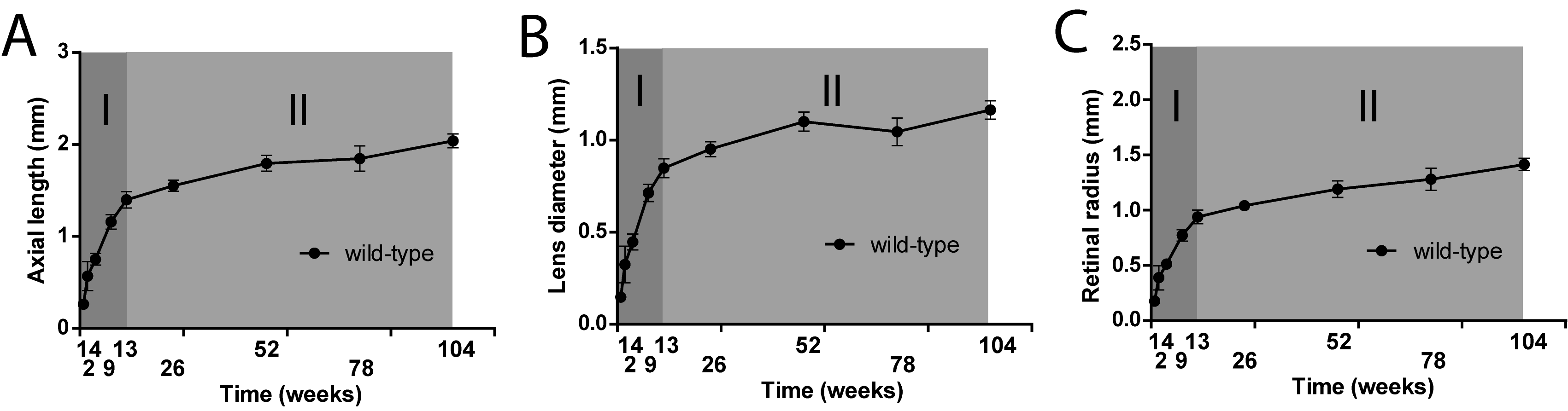

Supplement: Figure S2 — Zebrafish eye and body parameters graphed with respect to time using a linear X-axis for time. A. Eye axial length measured by SD-OCT increases during the lifetime of the growing wild-type fish in two phases. The first is rapid (labeled I, dark grey box, slope = 0.01326±0.0003736 (1/slope = 75)) and the second is slower (labeled II, light grey box, slope = 0.0009584±4.517e-005 (1/slope = 1043)). B. Lens diameter increases as the fish grows in two phases. The first is rapid (labeled I, dark grey box, slope = 0.1602±0.009701 (1/slope = 120)) and the second is slower (labeled II, light grey box, slope = 0.0004333±3.207e-005 (1/slope = 2308)). C. Retinal radius increases as the fish grows in two phases. The first is rapid (labeled I, dark grey box, slope = 0.008863±0.0002589 (1/slope = 113)) and the second is slower (labeled II, light grey box, slope = 0.0007279±3.085e-005 (1/slope = 1374)). (TIF) [file pone.0110699.s002.tif]

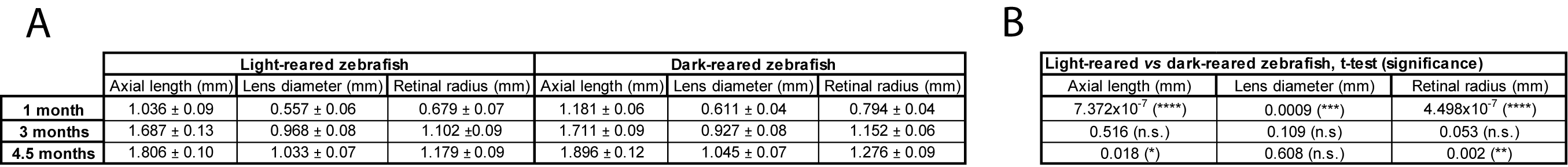

Supplement: Table S1 — A. Comparison of axial length, lens radius and retinal radius at 1 month, 3 months and 4.5 months between light-reared and dark-reared zebrafish. Values show average measurements ± SD. B. t-test results comparing axial length, lens radius and retinal radius for significant differences. (TIF) [file pone.0110699.s003.tif]
